# A machine learning analysis of the relationship of demographics and social gathering attendance from 41 countries during pandemic

**DOI:** 10.1038/s41598-021-04305-5

**Published:** 2022-01-14

**Authors:** Barnabas Szaszi, Nandor Hajdu, Peter Szecsi, Elizabeth Tipton, Balazs Aczel

**Affiliations:** 1 Institue of Psychology, ELTE Eötvös Loránd University, Budapest, Hungary; 2 Doctoral School of Psychology, Institute of Psychology, Eötvös Loránd University, Budapest, Hungary; 3 Northwestern University, Evanston, USA

**Keywords:** Human behaviour, Psychology, Diseases, Risk factors

## Abstract

Knowing who to target with certain messages is the prerequisite of efficient public health campaigns during pandemics. Using the COVID-19 pandemic situation, we explored which facets of the society—defined by age, gender, income, and education levels—are the most likely to visit social gatherings and aggravate the spread of a disease. Analyzing the reported behavior of 87,169 individuals from 41 countries, we found that in the majority of the countries, the proportion of social gathering-goers was higher in male than female, younger than older, lower-educated than higher educated, and low-income than high-income subgroups of the populations. However, the data showed noteworthy heterogeneity between the countries warranting against generalizing from one country to another. The analysis also revealed that relative to other demographic factors, income was the strongest predictor of avoidance of social gatherings followed by age, education, and gender. Although the observed strength of these associations was relatively small, we argue that incorporating demographic-based segmentation into public health campaigns can increase the efficiency of campaigns with an important caveat: the exploration of these associations needs to be done on a country level before using the information to target populations in behavior change interventions.

## Introduction

When there is no medical treatment available, the best way to address an unfolding epidemic is to convince people to adopt behavior patterns that can alleviate the spread of the disease^1,2^. Different protective behaviors, such as the avoidance of social gatherings, have been identified as an effective tool to decrease the spread of viruses^3–8^. Therefore, it has central importance in times of epidemics that these protective behaviors are widely adopted. In this study, we used the COVID-19 pandemic situation to explore the demographic groups that are the most likely to attend social gatherings in order to support public-health officials and policymakers to design targeted and more efficient campaigns during epidemic emergencies.

Knowing who to target is the prerequisite of quick and efficient public health campaigns. Having information on the key populations enables policymakers to design interventions that can take into account the specific context and the characteristics of the target group. Compared to group-tailored messaging, ‘one-fit-all’ interventions ignore the diversity of the populations, therefore, they are expected to be less efficient, potentially at the cost of human lives^9^. Taking a step further, it is possible that during pandemics, the same behavioral intervention has opposite effects on different populations. For example, the same campaign can increase adherence behavior in one group and motivate non-adherence in another (e.g.^10^) leading to avoidable death. As previous research suggests that the majority of people comply with the social distancing recommendations^11^, it is crucial that policymakers only target public health campaigns on those groups whose behavior needs to be changed.

Information on the psychological and sociological attributes (such as the values, norms or risk-perception) of individuals could be used to predict preventive behaviors (e.g.^12–14^) However, in the majority of the communications platforms (e.g., social media, television, radio, print), these pieces of information are not available, and public health officials can only use basic demographics for targeting public health campaigns and policies. For that reason, in the present work, we focus on age, education, gender, and income as they are the ones widely accessible to use.

Policymakers recommended or mandated several preventive behaviors such as mask-wearing, physical and social distancing, and handwashing^15^. In the empirical part of the present paper, we focus on a widely recommended e.g.^16^^,^^17^ but understudied protective behavior: individuals' avoidance of social gatherings. However, as prior published research involving the investigation of demographic factors did not specifically focus on the attendance of social gatherings, here we summarize the findings more generally focusing on the association of demographics and protective behavioral patterns.

Preventive behaviors have been found to vary along demographic factors (for a review see^15^). However, the direction and the strengths of these relationships showed a mixed picture. Age has been positively associated with preventive behaviors during pandemics in some studies (e.g.^16–19^), and negatively in others^20^, while some studies found no association (e.g.^21,22^). Income is often correlated with complying behavior^21,23–26^, a result which is argued to be found because low-paid workers are less able to work remotely and stay at home without losing their jobs. Studies investigating the relationship of gender and protective behavior also found mixed results: although most studies found that men are less likely than women to adhere to the protective recommendations (e.g.^19,27–29^), other studies showed evidence for the opposite^16^ or found no association^18^. Educational level has also been associated with opposing results: while some studies showed that higher education predicts more precautionary behavior^30,31^, others found contrary evidence^22^ or mixed results^19^. The variance of these results might be due to the fact that the preventive measures discussed are diverse (e.g., avoiding social gatherings, compliance with quarantine, or mask use) and different levels of adherence is expected for these different health behaviors. Furthermore, the studies typically had small sample sizes, with the samples coming from one or a small number of countries with diverse populations. .

In the present research, extending previous results, we explore the association between demographic factors and one specific behavior, the avoidance of social gatherings, and we investigate our question in a large sample (n > 80,000) collected from 41 countries, which makes the results more generalizable and comparable across different cultures. We use a machine-learning technique which allows us to identify not just the main effects of the demographic factors but also reveal subtle patterns and explore the heterogeneity between countries. We also discuss how these results can be used to improve public health interventions.

## Methods

### Dataset

In the present paper, we re-analyze the data collected by an international research collaboration during the early phase of the COVID-19 pandemic between 2020.03.20. and 2020.04.05.^32^. The data were gathered via snowball method using an online survey; participants were recruited from all over the world via an open call spread through social media and several media outlets. As a result, 112,136 individuals filled out the survey from 175 countries. To be eligible to participate in the study, volunteers had to accept the informed consent and be at least 18 years old. The detailed method of data collection, the full list of survey items and their order can be read in Fetzer and his colleagues’ paper^32^. Note, that Fetzer et al. collected a broad list of items, but here we discuss the ones relevant for the present study.

From this dataset, we excluded the responses of individuals who did not complete the full survey. Furthermore, we included responses only from those individuals who lived in a country at the time of survey completion where there was a coordinated public information campaign on COVID-19 targeting the general population (and not just a subpopulation). To do so, we used the data from the Oxford COVID-19 Government Response Tracker 35, which collects publicly available information on COVID-19 related governmental responses in each country. Furthermore, the data of those who reported nonsensical values: age over 99 years and households with 0 members were removed, along with those who reported their years of education being either under 4 or more than their age minus 5. Finally, to maximize the reliability of the survey in each country, responses from countries with fewer than 400 respondents were also not analyzed. As a result, our final dataset consisted of 87,169 individual responses from 41 countries (56% female, *M*_*age*_ = 40.0, *SD*_*age*_ = 12.8) with 2126 mean number of respondents per country. As of 2020, these countries accounted for 73.05% of the world’s population. (World population estimation was based on the UN’s World Population Prospects, accessed from Word Population Review)^33^. A detailed description of the sample in each country can be found in Supplementary Table 1. The data are available at the projects’ OSF page: https://osf.io/rehc7/.

### Procedures and measures

As part of a broader online data collection effort^32^, participants responded to several COVID-19 related survey items. Crucially, for the purposes of this study, respondents were asked to indicate on a 100-point scale to what extent the statement ‘*I did not attend social gatherings*’ describes their behavior for the past week. This item was our key measure assessing individuals’ behavior regarding social gatherings. We categorized individuals who indicated total agreement (*100 points*) with the statement that they did not attend social gatherings as *social gathering avoiders*, while the rest of the participants were classified as *social gathering goers*. We decided to dichotomize the social gathering variable, as in our dataset, a substantial number of values were not represented for many countries which could otherwise lead to unwanted bias in the models.

Furthermore, participants responded to several questions regarding their demographics including age (*Which year were you born?),* gender (*Which gender do you identify with? Male; Female; Other*), education (*How many years of education did you complete?*), country of residence (*In which country do you mostly live?*). Participants also indicated their household income (*What is your monthly household income, before tax, in your country’s currency?*) and their household size *(How many people live in your household?*). Following previous recommendations^34^, we used adjusted household income in our analyses. Adjusted household income was calculated by dividing household income by the square root of household size.

### Data-analysis strategy

To explore the role of demographics in the avoidance of social gatherings, random forest models were applied as they handle unbalanced data relatively well compared to logistic regression models^35^. Random forests are used extensively in machine learning either for regression or classification problems because they are robust to the non-linearity of data, they do not require data to be normalized, and they mitigate overfitting without extensive parameter tuning. Random forest models operate by creating decision trees. Each decision tree in the forest consists of a set of internal nodes and leaves. In the internal node, a feature is selected along which the data is split into two groups. Each group is then iteratively subdivided according to the same rules until a condition regarding the size of the tree or the number of data points in the node is met. Information gain was chosen as the criterion for selecting features in our calculations. The average increase in information gain is collected for each selected variable. Then, the mean of this increase over all trees in the forest is calculated; this is the measure of variable importance. The resulting tree is random, because random subsets of the variables are used for the trees. But, with many trees, the resulting importance values should be similar.

Instead of fitting a global model on the overall population, we fit individual models to each country, as the disease progression, policy measures, and political and public health messaging—as well as more general social and behavioral norms—vary dramatically from country to country in ways that are difficult to appropriately adjust and control for. Moreover, using country-specific models enables us to explore the heterogeneity across countries. Note that although we access a relatively large sample from each country, we re-weight the observations based on the respondent’s gender, age, income, and education in the main analyses to make the collected data more representative at the country level.

In our analyses, we split data from each country into training and test sets in an 80–20 ratio, and we use the training set to find the number of variables sampled at each split of a decision tree for our random forest models. To search for the optimal number of random variables to select at each split, we used repeated tenfold cross-validation, tuned separately for every country. This resampling procedure divides the training sample into 10 subsets, a.k.a. folds, and uses 9 of them to train the model and the remaining subset to validate its accuracy. This procedure is repeated 10 times, with a different subset left out each time as a validation set^36^. Then, the final model metrics are calculated by averaging the metrics of 10 previously trained models. Finally, the models developed through the tenfold cross-validation were tested on the test sample. Because the number of social gathering goers (24%) and avoiders (76%) is unequal in our dataset, we upsample the training data; this means that we sample with replacement from the original, minority class data until we reach a sample size equal to the majority class. This way, in the training data, social gathering avoiders and goers are balanced. From the many well-established accuracy metrics, we chose to tune our models on the training set to get the greatest area under the precision-recall curve (prAUC), because it is fairly robust to unbalanced data. Finally, we use the test set to see how well each previously trained model performs. The analysis code is available at https://osf.io/rehc7/.

## Results

We created and ran random forest models for each country with the specifications detailed above. The models successfully predicted attendance of social gatherings based on demographic factors during the early phase of the pandemic but also showed significant cross-country heterogeneity ranging from 0.52 to 0.84. For a detailed description of the models and prediction accuracies see Supplementary Table 2.

### The association between the demographic factors and the avoidance of social gatherings across countries

First, to explore the association of demographic variables and the avoidance of social gathering across the world, we calculated descriptive statistics on the proportion of social gathering goers and avoiders in each country in the following subgroups: female participants, male participants; individuals reporting lower than the median income, higher than the median income; lower than median age, higher than median age; lower than median education, and higher than median education. Based on this categorization, we found that the proportion of social gathering goers was higher for males than females in 95% of the countries (39 countries), among low-income than high-income people in 80% of the countries (33 countries), among younger than older people in 78% of the countries (32 countries), and among lower educated than higher educated people in 66% of the countries (27 countries).

**Figure 1:**
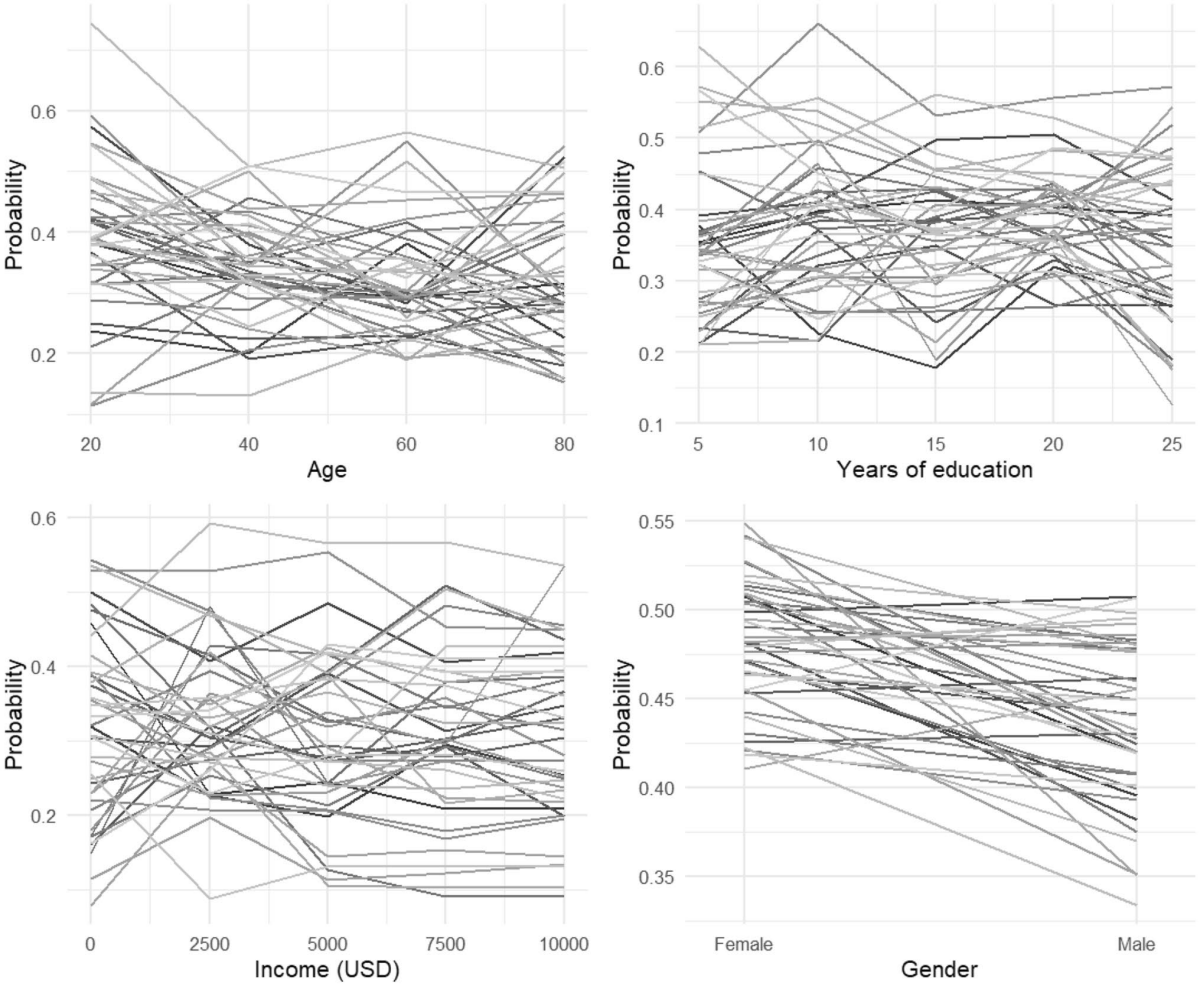
Partial dependence plots show the average predicted probability of attending social gatherings associated with a given value of the demographic factor of age, years of education, income, and gender (in different plots) for all the countries.

Next, using the results from the random forest models, we created partial dependence plots in order to see whether each of the demographic factors were associated with higher or lower probability of social gathering avoidance (Fig. 1). Partial dependence plots show the average predicted probability of leaving home associated with a given value of the demographic factor in each country. Plotting these lines on the same graph for each country makes it possible to recognize mutual trends in the change of probabilities and explore the heterogeneity of the results. Accordingly, Fig. 1 shows that in most of the countries, being at older age, being female, having a lower income, and more years of education seem to indicate a lower probability of attending social gatherings, although there is significant heterogeneity across countries for each of the demographic variables except gender.

### General importance of demographic factors at predicting the avoidance of social gatherings

We also calculated variable importance scores for each demographic factor in each country. The variable importance score is a metric expressing the mean increase in accuracy when a given variable is added to a model, that is, it shows how important a variable is at improving the overall predictive power of a model with the other parameters keeping constant.

**Figure 2:**
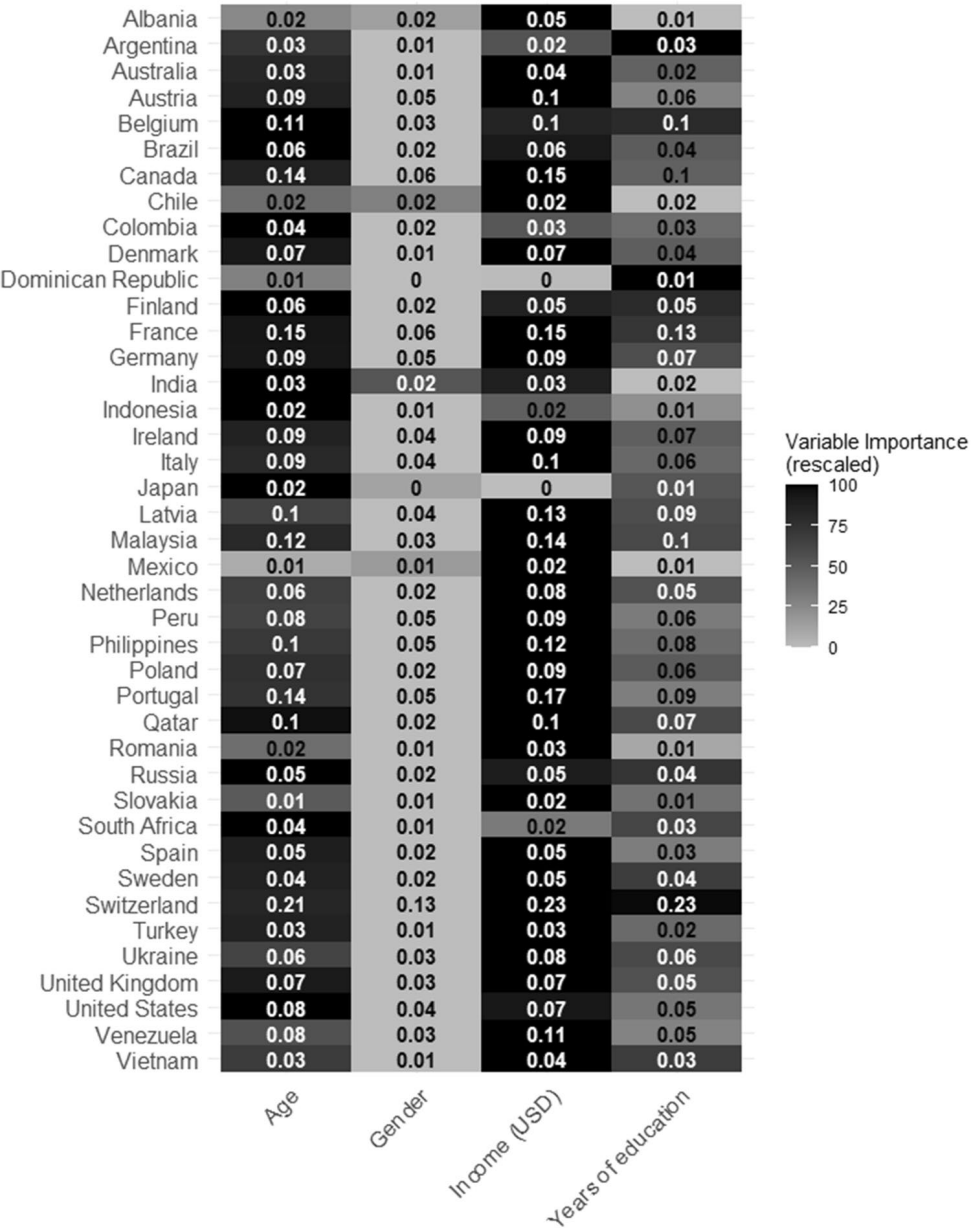
The figure summarizes the variable importance scores for each demographic variable in each country. Variable importance values express the mean increase in accuracy when a given demographic variable is added to a model. The coloring of the figures depicts the relative importance of the variables within each country while the variable importance values were rescaled between 0 and 100 in each country, 100 being the most (darkest) and 0 being the least important (lightest).

The median importance score of income was 0.07 (with a range of 0–0.23), meaning that adding information about income would make our predictions around 7% more accurate. This value was 0.05 (with a range of 0–0.21) in the case of age, 0.05 (with a range of 0–0.23) in the case of education, and 0.02 (with a range of 0–0.13) in the case of gender. Figure 2 summarizes the variable importance scores for each demographic factor in each country.

### Relative importance of demographic factors at predicting the avoidance of social gatherings

We determined the strongest predictor of social gathering avoidance by determining the demographic factor with the greatest variable importance score in each country. Out of the 41 countries examined, the strongest predictor was income in 29 countries, age in 10 countries, and education in 2 countries. The variable with the lowest importance score was gender in 36 countries, years of education in 4 countries, and income in 1 country (see Fig. 1).

## Discussion

To explore which demographic subgroups are the most likely to visit social gatherings during epidemic emergencies, we investigated a large dataset collected during the early phase of the COVID-19 pandemic situation from 41 countries. With these countries accounting for 73.05% of the world’s population, our study provides the first global, systematic investigation of association between demographic factors’ and individuals' tendency to attend or avoid social gatherings during pandemics.

While prior studies showed mixed results, we found a few general patterns arising: in the majority of the countries, the proportion of social-goers was higher in male than female, younger than older, lower-educated vs. higher educated, and low-income vs. high-income subgroups of the population. However, we also observed noteworthy heterogeneity among the countries regarding the direction of the association of the investigated demographic factors and the propensity to visit social gatherings. For example, in 33% of the countries, high-educated citizens were more prone to non-adhere to the avoidance of social gatherings than their low-educated counterparts. Such heterogeneity warrants policymakers and researchers to simply generalize such behavior from one country to another without understanding the specific context, and suggests that simply targeting older, low-income, low-educated, male citizens in public health campaigns is not a proper solution.

When resources are tight and the targeting of an intervention needs to be made based on one given demographic variable, one needs to know which variable this should be^37^. Relative to the other demographic factors, our results provided evidence that income was the strongest predictor worldwide when it comes to visiting social gatherings followed by age, education, and finally gender, but again we found large heterogeneity between the countries. Even countries that are geographically and culturally close (such as Germany and Austria) showed different patterns**.** One potential reason behind this variability is that the identification of the strongest demographic predictor can be sensitive to the correlation among the demographic variables and it also fails to account for synergistic effects between two or more demographic factors^38^. That is, instead of finding an emerging trend across countries, the results confirmed that the investigated associations are heterogeneous, largely differing from country to country. Such findings bring evidence that context has a non-ignorable moderating power on the relationship of social gathering behavior and demographic factors, and they suggest that the exploration of this association needs to be done on a country-level.

Although the observed strength of the associations between the demographics and avoidance of social gatherings are often small, these small effects can have meaningful and important consequences. When analyzing the country-level data, we found for example that across the investigated countries, the youngest 20% of the population were on average ~ 4% more likely not to adhere to the avoidance of social gathering than the oldest 20% of the population (see Supplementary Materials). Note, that this is a sizable difference. Previous evidence suggests that every 1% increase in non-essential visits lead to 7–8% increase in new COVID-19 cases the following week^39^.

Targeting these less avoidant demographic subgroups of the population with public health campaigns could have important advantages. First, affecting only those subgroups who need to be affected could save public resources and decrease the risk of the potential conflicting effects on adherent subgroups. Note, that we also found that 75% of our sample reported absolute avoidance of social gatherings, and these populations don’t need to be addressed by prevention campaigns. Second, the identification of non-adherent groups would enable intervention designers to increase the effectiveness of public health campaigns by tailoring the messages to the target population-specific habits, beliefs, and attitudes.

The investigation of the moderating factors behind the revealed patterns would be an important avenue for future research. One of these factors is that countries apply different policies regarding how citizens should behave during the pandemic^40^. Although we only included countries in our analysis where there was a public information campaign about COVID-19, the recommendations and regulations were diverse across countries which could also affect the attendance of social gatherings. For instance, at the time of the data collection of the present research, the UK suggested that individuals do not leave their houses, except for very limited purposes, e.g., shopping for basic necessities, exercise once a day, any medical need, and travelling for work purposes where working from home is impossible. Similarly, public transportation was more restricted in Albania, Dominican Republic, India, the Philippines, and Qatar compared to other countries in our dataset. Beyond regulations, perceived and self-efficacy^41^, cultural differences (e.g.^42^), higher tendency to avoid uncertainty^43^, trust in government^44,45^ or trust in a higher power^46^ have also been shown to explain disparities between different populations. Furthermore, differences in the proportion of blue-collar jobs could also explain these differences, as job type has been shown to be associated with differential levels of social distancing^47,48^. However, the list of potential factors is much longer and needs a systematic investigation itself.

Further studies could also explore how the conceptualizations of the demographic variables affect the results. For example, we calculated the ‘income’ variable by dividing the reported household income by the square root of household size, because we aimed to adjust for the fact that the needs of a household with each additional member do not grow in a proportional way. Alternative approaches focusing on different aspects of the question such as using the total household income, dividing the household income by the total number of people in the household, or using other kinds of equivalence scales (for a review, see^49^) may have yielded different results.

The present study has several limitations. First, our data was collected during the early phase of the pandemic, and it is possible that the adherence of different demographic groups changed in its later stages. Although our data are not suitable to resolve this concern, a longitudinal study found that the strength of association between different demographic groups and social distancing was similar from April to August 2020^19^. Second, the data used in the present research are based on self-reports. The results from Gollwitzer et al.^50^ suggest that this is not necessarily a problem: the authors connected self-reports with 17 million smartphone GPS coordinates during the COVID-19 pandemic and found that self-reported data followed actual social distancing behavior. Third, it needs to be noted that pandemics may vary in features driving decisions regarding the attendance of social gatherings (e.g., different death rates between different demographic groups), therefore, our findings may not generalize to all future pandemics. Fourth, our study focused on the association of demographic variables and attendance of social gatherings but did not provide causal explanations behind the observed patterns. Future studies addressing why the revealed associations emerge (e.g., differences in home working opportunities or housing conditions, perceptions, beliefs), might be able to explain some of the variance observed across the countries.

## Supplementary Information

Supplementary Information.

## Data Availability

The authors declare that all methods were performed in accordance with the relevant regulations. All the data and analysis code of this project are available from https://osf.io/rehc7/. The transparency report^51^ of the project is available from https://osf.io/f3nug/.
