# Supplementary material for: A machine learning analysis of the relationship of demographics and social gathering attendance from 41 countries during pandemic
Source: Sci Rep. 2022 Jan 14;12:724. doi: 10.1038/s41598-021-04305-5 (PMC8760248; doi:10.1038/s41598-021-04305-5)

**Supplementary Materials**

Table 1. Descriptive Statistics Per Country

| **Country** | **N** | **Ratio of females** | **Ratio of social gathering goers** | **Median adjusted household income (USD)** | **Adjusted household income SD** | **Median age** | **Age SD** | **Median years of education** | **Years of education SD** |
| --- | --- | --- | --- | --- | --- | --- | --- | --- | --- |
| Albania | 671 | 0.64 | 0.21 | 486.19 | 1074.42 | 34 | 9.59 | 17 | 2.79 |
| Argentina | 807 | 0.43 | 0.11 | 965.63 | 1202.35 | 37 | 11.59 | 18 | 4.21 |
| Australia | 888 | 0.67 | 0.39 | 4735.95 | 33098.35 | 42 | 13.04 | 17 | 3.36 |
| Austria | 1056 | 0.53 | 0.15 | 3098.82 | 11958.12 | 38 | 11.87 | 17 | 3.56 |
| Belgium | 512 | 0.54 | 0.12 | 3795.27 | 19504.84 | 37 | 10.73 | 17 | 5.56 |
| Brazil | 9351 | 0.61 | 0.23 | 867.35 | 1315.97 | 34 | 13.1 | 17 | 4.44 |
| Canada | 2615 | 0.65 | 0.14 | 9985.2 | 32117.46 | 43 | 13.3 | 18 | 4.32 |
| Chile | 503 | 0.55 | 0.21 | 2008.74 | 2090.62 | 40 | 11.21 | 18 | 3.55 |
| Colombia | 1682 | 0.48 | 0.23 | 1107.06 | 1640.68 | 36 | 11.01 | 18 | 4.23 |
| Denmark | 422 | 0.51 | 0.24 | 3612.84 | 14322.23 | 37.5 | 11.91 | 17 | 5.38 |
| Dominican Republic | 472 | 0.52 | 0.19 | 1575.79 | 4212.44 | 37 | 10.51 | 19 | 4.66 |
| Finland | 725 | 0.54 | 0.29 | 4260.88 | 26246.69 | 40 | 11.5 | 17 | 5 |
| France | 2067 | 0.53 | 0.11 | 3162.72 | 18509.87 | 36 | 13.04 | 7 | 6 |
| Germany | 9686 | 0.49 | 0.24 | 3286.8 | 18844.17 | 37 | 11.8 | 17 | 3.83 |
| India | 837 | 0.39 | 0.23 | 1038.16 | 8087.86 | 33 | 10.67 | 18 | 3.19 |
| Indonesia | 1417 | 0.56 | 0.4 | 176.38 | 650.45 | 27 | 7.98 | 16 | 3.22 |
| Ireland | 668 | 0.49 | 0.13 | 4648.24 | 26597.29 | 41 | 10.59 | 18 | 3.61 |
| Italy | 1744 | 0.47 | 0.09 | 2739 | 18386.98 | 37 | 13.15 | 18 | 3.39 |
| Japan | 556 | 0.41 | 0.48 | 5311.41 | 46030.04 | 45 | 12.52 | 18 | 3.06 |
| Latvia | 582 | 0.72 | 0.14 | 1095.6 | 1163.72 | 34 | 9.43 | 17 | 3.34 |
| Malaysia | 480 | 0.58 | 0.17 | 1150.44 | 4636.23 | 38 | 12.17 | 17 | 3.93 |
| Mexico | 860 | 0.53 | 0.27 | 1052.59 | 2225.67 | 42 | 12.34 | 19 | 3.54 |
| Netherlands | 1297 | 0.57 | 0.23 | 3834.6 | 17829.14 | 40 | 11.8 | 18 | 3.83 |
| Peru | 1835 | 0.42 | 0.11 | 1066.85 | 2899.59 | 38 | 11.19 | 17 | 4.09 |
| Philippines | 621 | 0.68 | 0.13 | 630.62 | 1973.91 | 31 | 10.34 | 16 | 3.21 |
| Poland | 462 | 0.56 | 0.22 | 1364.22 | 9089.57 | 35 | 10.19 | 17 | 3.24 |
| Portugal | 537 | 0.69 | 0.11 | 1095.6 | 6320.85 | 37 | 10.97 | 17 | 3.35 |
| Qatar | 1016 | 0.7 | 0.27 | 3071.52 | 7234.01 | 29 | 11.5 | 16 | 3.8 |
| Romania | 788 | 0.65 | 0.27 | 1575.2 | 3125.23 | 37 | 8.96 | 17 | 2.75 |
| Russia | 3110 | 0.4 | 0.35 | 668.47 | 933.16 | 33 | 10.64 | 15 | 2.6 |
| Slovakia | 597 | 0.53 | 0.34 | 1643.4 | 1210.55 | 34 | 9.13 | 17 | 2.64 |
| South Africa | 496 | 0.71 | 0.3 | 1778.54 | 8793.5 | 42 | 12.13 | 16 | 4.27 |
| Spain | 2089 | 0.5 | 0.11 | 2324.12 | 15981.47 | 44 | 11.91 | 20 | 4.69 |
| Sweden | 5461 | 0.69 | 0.62 | 3070.49 | 7183.65 | 46 | 12.16 | 16 | 3.9 |
| Switzerland | 3486 | 0.55 | 0.13 | 6592.05 | 39142.67 | 41 | 11.85 | 15 | 5.65 |
| Turkey | 2773 | 0.53 | 0.25 | 459.99 | 822.17 | 31 | 9.75 | 17 | 2.84 |
| Ukraine | 1367 | 0.72 | 0.21 | 377.98 | 593.33 | 28 | 9.39 | 15 | 2.92 |
| United Kingdom | 10550 | 0.51 | 0.26 | 6992.15 | 32891.84 | 43 | 12.52 | 17 | 3.52 |
| United States | 10686 | 0.61 | 0.18 | 18500 | 54564.77 | 40 | 13.17 | 18 | 3.41 |
| Venezuela | 626 | 0.45 | 0.16 | 52.21 | 1482.15 | 54 | 12.26 | 18 | 4.24 |
| Vietnam | 771 | 0.77 | 0.31 | 345.5 | 759.73 | 21 | 6.25 | 13 | 2.84 |

To get a comparable estimate of household incomes across different countries, we converted the declared income data to USD. As an exchange rate, we used the national currency per U.S. end of month dollar rate of March 2020 ^52^.

Table 2. Parameters of the tuned models and accuracy on test data.

| **Country** | **No. of vars sampled at each split** | **prAUC** | **Accuracy** |
| --- | --- | --- | --- |
| Albania | 2 | 0.977 | 0.617 |
| Argentina | 2 | 0.979 | 0.820 |
| Australia | 2 | 0.979 | 0.520 |
| Austria | 2 | 0.979 | 0.738 |
| Belgium | 2 | 0.978 | 0.802 |
| Brazil | 2 | 0.977 | 0.640 |
| Canada | 2 | 0.977 | 0.711 |
| Chile | 2 | 0.977 | 0.700 |
| Colombia | 2 | 0.976 | 0.627 |
| Denmark | 2 | 0.978 | 0.655 |
| Dominican Republic | 2 | 0.978 | 0.713 |
| Finland | 2 | 0.979 | 0.531 |
| France | 2 | 0.978 | 0.772 |
| Germany | 2 | 0.980 | 0.605 |
| India | 2 | 0.975 | 0.663 |
| Indonesia | 2 | 0.977 | 0.572 |
| Ireland | 2 | 0.979 | 0.722 |
| Italy | 2 | 0.970 | 0.842 |
| Japan | 2 | 0.974 | 0.532 |
| Latvia | 2 | 0.975 | 0.696 |
| Malaysia | 2 | 0.978 | 0.750 |
| Mexico | 2 | 0.978 | 0.673 |
| Netherlands | 2 | 0.976 | 0.667 |
| Peru | 2 | 0.976 | 0.730 |
| Philippines | 2 | 0.977 | 0.610 |
| Poland | 2 | 0.976 | 0.761 |
| Portugal | 2 | 0.979 | 0.802 |
| Qatar | 2 | 0.977 | 0.599 |
| Romania | 2 | 0.975 | 0.637 |
| Russia | 2 | 0.975 | 0.585 |
| Slovakia | 2 | 0.980 | 0.585 |
| South Africa | 2 | 0.980 | 0.626 |
| Spain | 2 | 0.977 | 0.734 |
| Sweden | 2 | 0.978 | 0.549 |
| Switzerland | 2 | 0.979 | 0.789 |
| Turkey | 2 | 0.976 | 0.596 |
| Ukraine | 2 | 0.982 | 0.621 |
| United Kingdom | 2 | 0.977 | 0.585 |
| United States | 2 | 0.980 | 0.666 |
| Venezuela | 2 | 0.978 | 0.750 |
| Vietnam | 2 | 0.979 | 0.569 |

Number of variables sampled at each split of a decision tree and area under the precision-recall curve of the tuned models by country. Accuracy represents the prediction accuracy of each tuned model on test data.

**Calculating the differences in avoidance of social gatherings between the top 20% and the bottom 20% of the population**

We also calculated the difference in avoidance of social gatherings between the top 20% and the bottom 20% for each demographic factor across the investigated countries. We observed that the youngest 20% of the population were on average 4.17% (with median of 5%) more likely not to adhere to social distancing than the oldest 20% of the population, while males were on average 4.07% (with a median of 4%) more likely not to adhere to social distancing than females. We found that across the investigated countries, the poorest 20% of the population were on average 0.48%(with a median of 1%) more likely not to adhere to social distancing than the richest 20% of the population while the most educated 20% of the population were on average 3.46% (with a median of 3%) more likely not to adhere to social distancing than the least educated 20% of the population.

Supplement Figure 1. Partial dependence plots showing the effect of ‘Age’ on attendance of social gathering, by country.
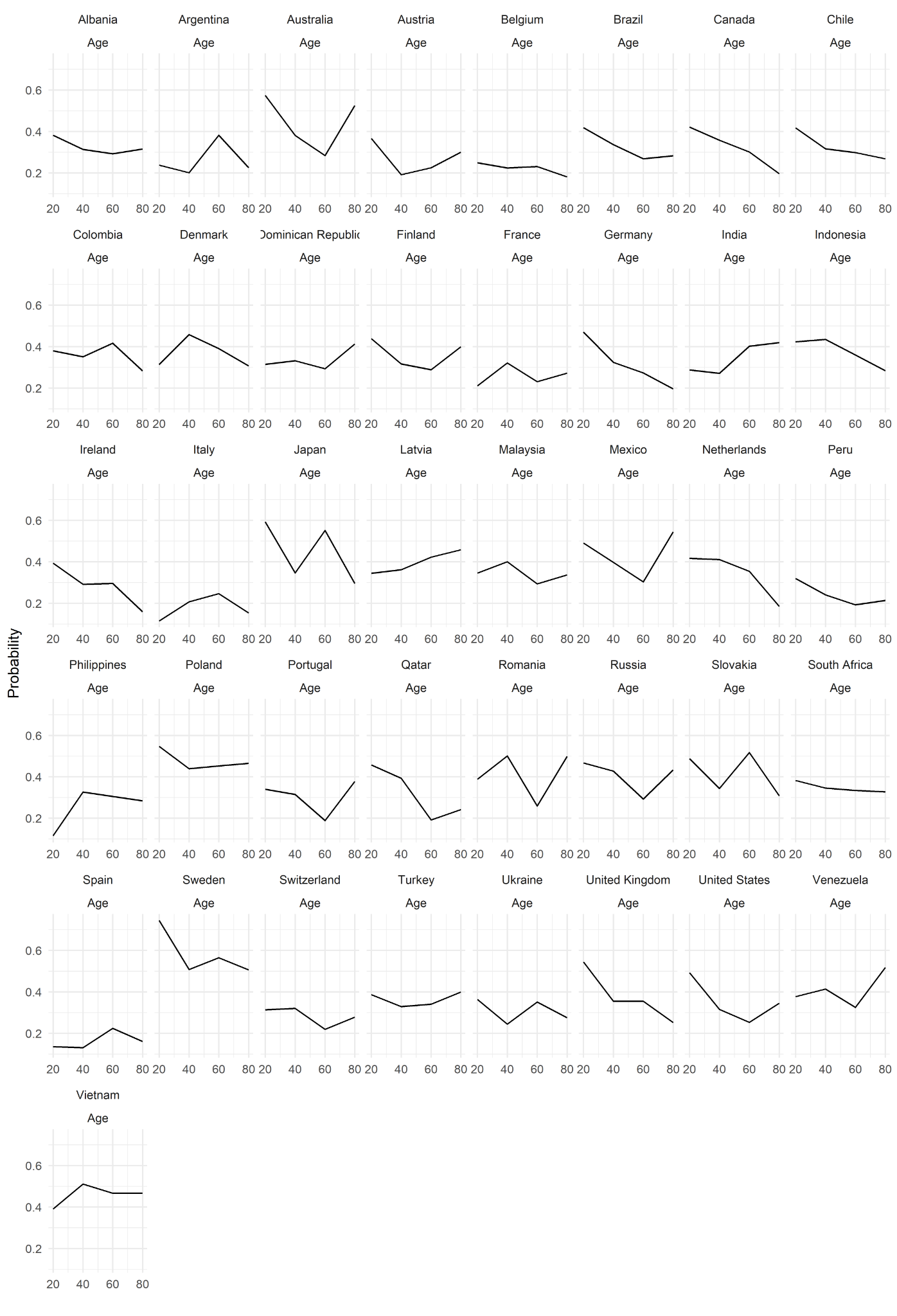


Supplement Figure 2. Partial dependence plots showing the effect of ‘Year of education’ on attendance of social gathering, by country.


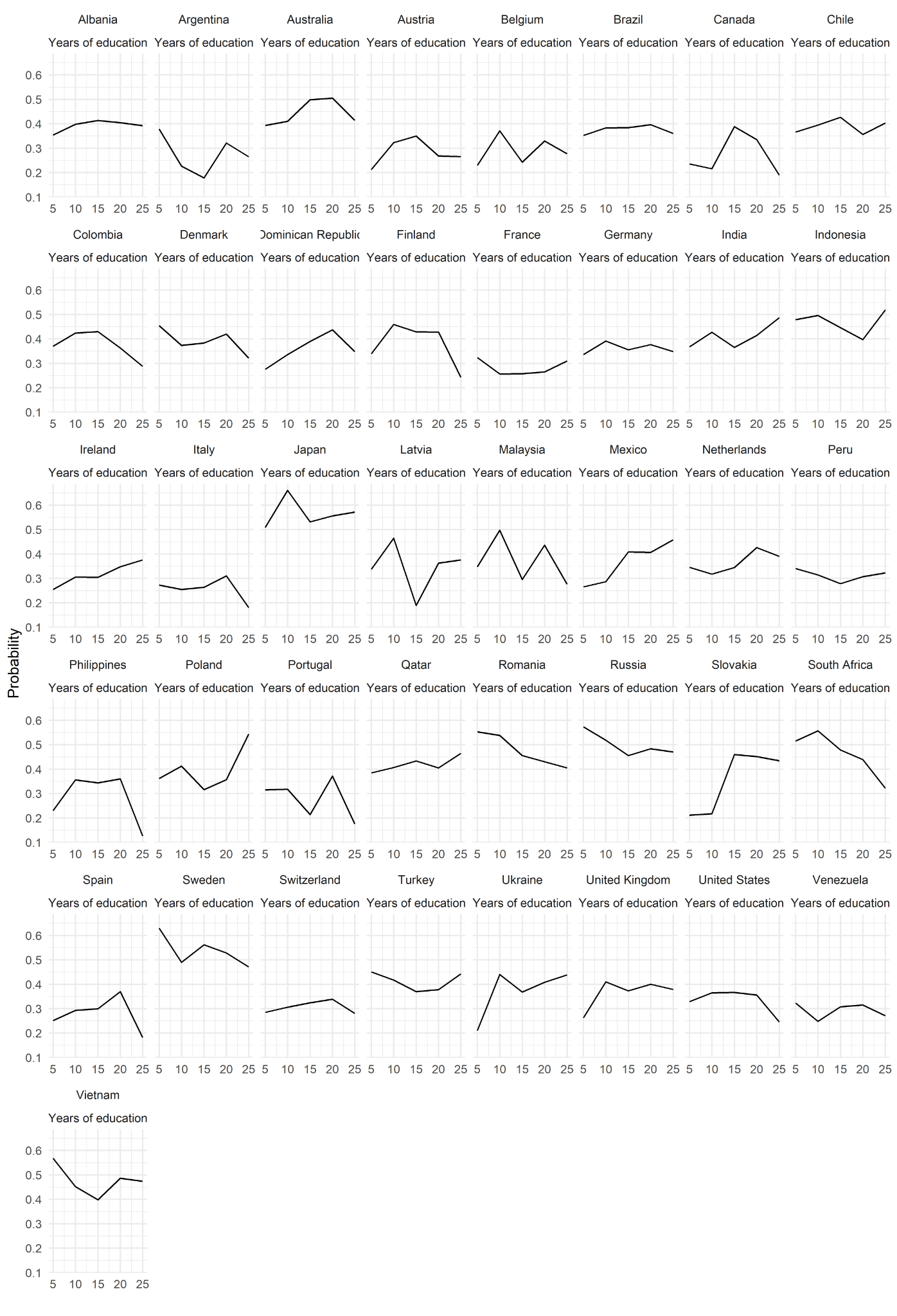


Supplement Figure 3.Partial dependence plots showing the effect of ‘Income’ on attendance of social gathering, by country.


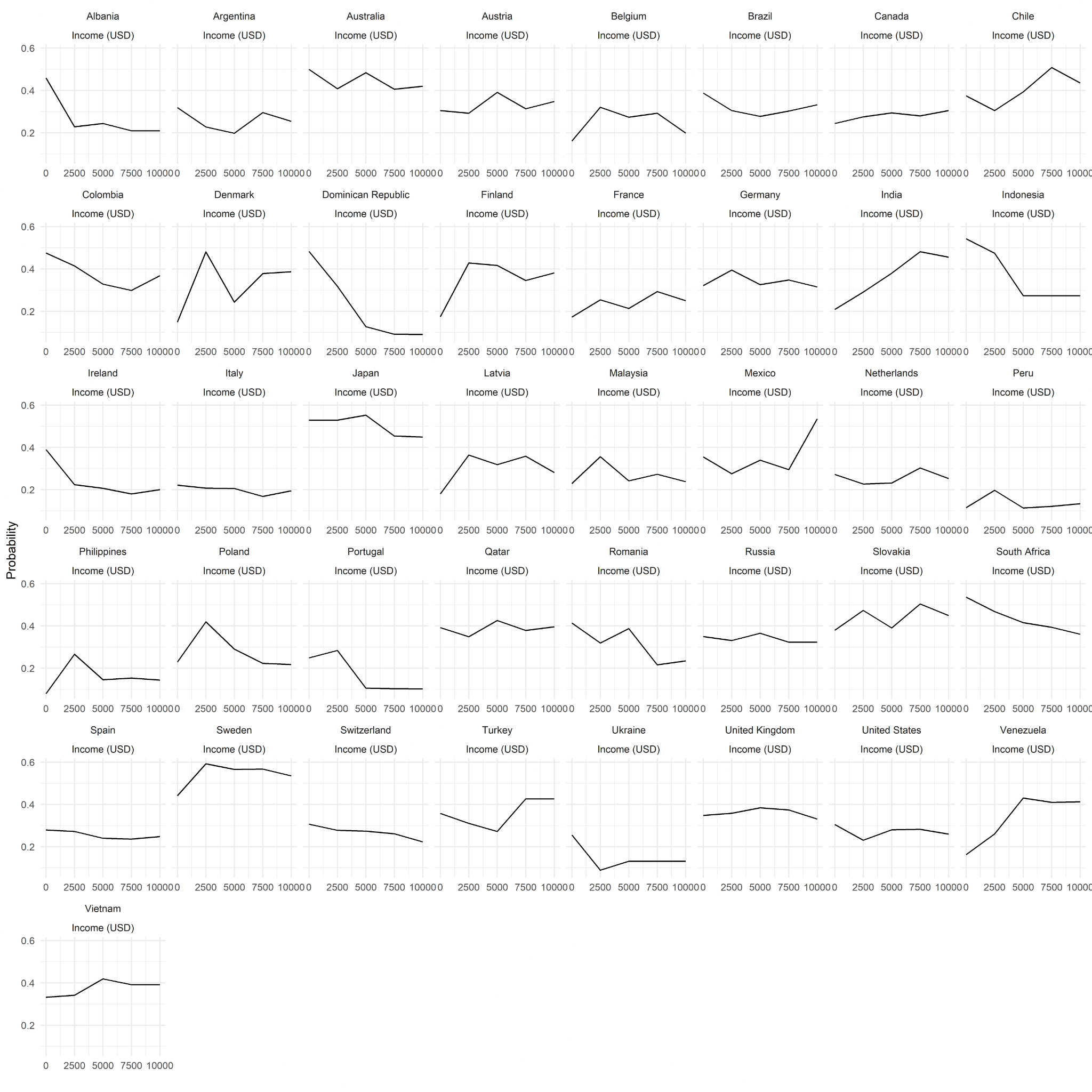


Supplement Figure 4. Partial dependence plots showing the effect of ‘Gender’ on attendance of social gathering, by country.


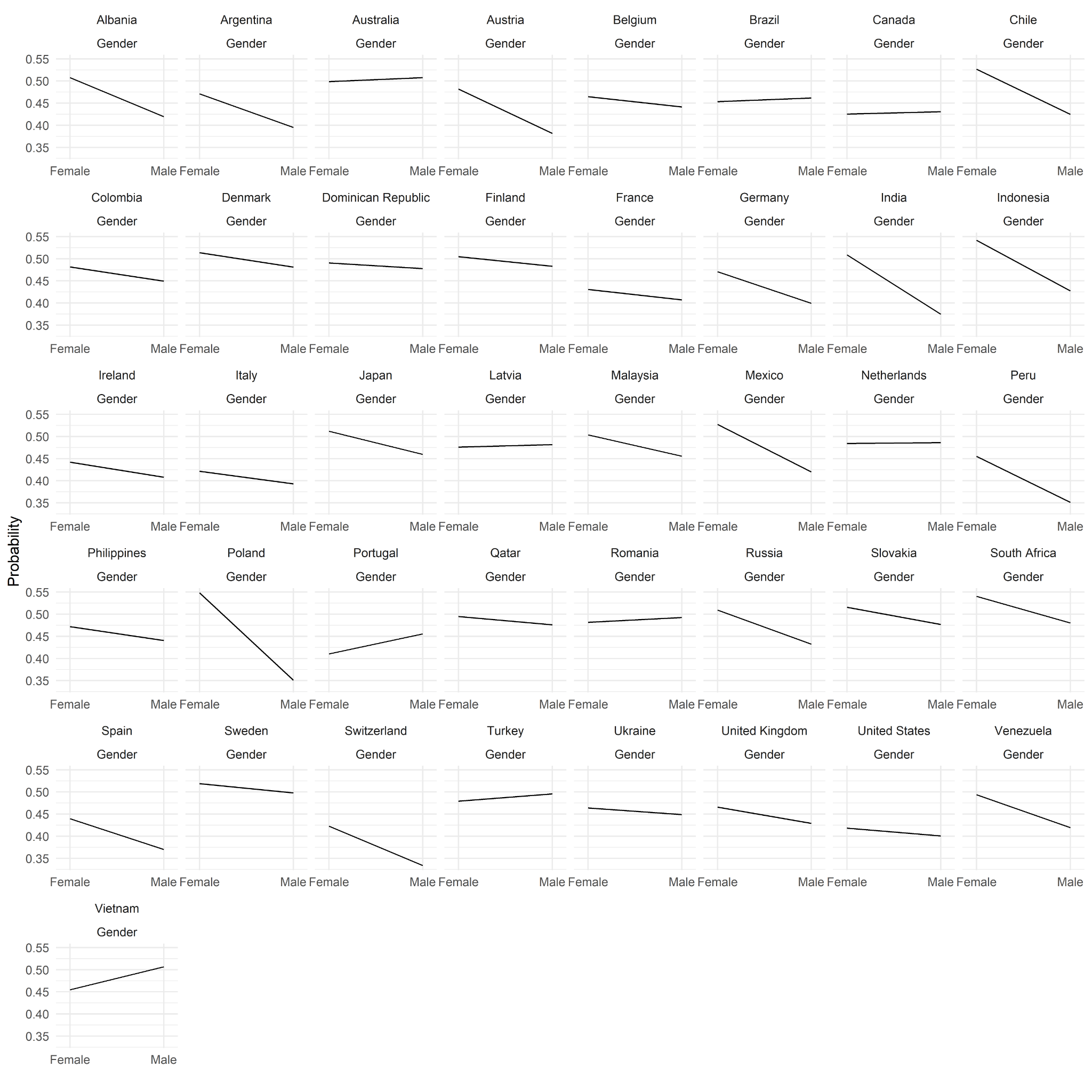

Supplement: Supplementary file 1 — Supplementary Information. [file 41598_2021_4305_MOESM1_ESM.docx]
